# SSZ‐13 Zeolite with Isolated Co^2+^ Sites as an Efficient and Durable Catalyst System for Non‐Oxidative Ethane Dehydrogenation

**DOI:** 10.1002/anie.202519600

**Published:** 2026-01-07

**Authors:** Qiyang Zhang, Tao Zhang, Bing Liu, Elizaveta Fedorova, Dmitry E. Doronkin, Evgenii V. Kondratenko

**Affiliations:** ^1^ School of Chemistry and Life Resources Renmin University of China Beijing 100872 P.R. China; ^2^ Department of Advanced Methods for Applied Catalysis Leibniz‐Institut für Katalyse e.V. (LIKAT) Albert‐Einstein‐Str. 29a 18059 Rostock Germany; ^3^ Department of Chemical Engineering, School of Chemical and Material Engineering Jiangnan University Wuxi 214122 P.R. China; ^4^ Institute for Chemical Technology and Polymer Chemistry, and Institute of Catalysis Research and Technology Karlsruhe Institute of Technology Kaiserstr. 12 D‐76131 Karlsruhe Germany

**Keywords:** Co/SSZ‐13, Co^2+^‐Z_2_ sites, Ethane dehydrogenation, Isolated Co^2+^ Ions, Structure–performance relationship

## Abstract

Non‐oxidative dehydrogenation of ethane (EDH) is an attractive method for on‐purpose ethene production, but achieving high activity and, especially, durability with catalysts based on earth‐abundant metals remains challenging. Herein, we introduce the Co/SSZ‐13 system with exclusively divalent cobalt (Co^2+^) ions that meets the above requirements. The use of complementary characterization techniques enabled us to reveal two Co^2+^ species: Co^2+^─Z_2_ located in the six‐membered‐ring windows and [Co(OH)]^+^─Z in the eight‐membered‐ring windows, with Z representing a charged zeolite framework site. A quantitative correlation between the rate of ethene formation and the site population establishes Co^2+^─Z_2_ as the active species. In situ X‐ray absorption spectroscopy confirms their structural and electronic stability under high‐temperature reaction conditions. The optimized 0.9Co/SSZ‐13 (0.9Co) catalyst showed highly durable operation over 200 dehydrogenation/oxidative regeneration cycles at 600–650°C lasting for 150 h with industrially relevant productivity. In this regard, it outperforms almost all previously developed catalysts even those with platinum as an active component. The obtained results uncover the atomic‐level origins of EDH activity/durability of the Co/SSZ‐13 system and highlight the critical role of metal site location in designing highly active, selective, and durable catalysts for on‐purpose ethene production.

## Graphical abstract

Catalysts with the isolated Co^2+^ species in the 6MR (Co^2+^─Z_2_) and 8MR ([Co(OH)]^+^─Z) windows of the SSZ‐13 zeolite were synthesized for non‐oxidative ethane dehydrogenation to ethene. The Co^2+^─Z_2_ species demonstrate an unexpectedly high rate of ethene formation in comparison with the ([Co(OH)]^+^─Z species. The most active developed catalyst with Co^2+^─Z_2_ outperforms analogues of commercial K‐CrO*_x_*/Al_2_O_3_ and PtSn/Al_2_O_3_ in terms of ethene formation.

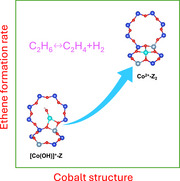

## Introduction

Ethene, the most broadly produced organic chemical in the modern chemical industry, is widely used for the manufacture of higher olefins, polyethene, ethene oxide, fibers, plastics, etc.^[^ ^1^, ^2^, ^3^ ^]^ The current large‐scale processes for ethene production are based on steam cracking of naphtha or steam cracking of ethane. The former process, with only 10%–35% ethene selectivity, is the most energy‐consuming in the petrochemical industry leading to copious carbon dioxide emissions.^[^ ^4^, ^5^ ^]^ Ethane cracking is also an energy‐demanding process and needs high reaction temperatures, above 800°C, due to its non‐catalytic nature. Nevertheless, the ethane‐based technology is more efficient in view of costs of ethene production.^[^ ^6^ ^]^

In the context of the challenges posed by current ethene‐producing technologies and large‐scale exploitation of shale gas,^[^ ^1^ ^]^ catalytic ethane dehydrogenation may revolutionize large‐scale ethene production. Both non‐oxidative and oxidative dehydrogenation reactions are currently under investigation,^[^ ^1^, ^2^ ^]^ with the latter route being more favorable from a thermodynamic point of view. However, the non‐oxidative ethane dehydrogenation (EDH) appears to be more promising because of higher selectivity and easier feed preparation and product separation. Although supported Pt‐ or Cr‐containing catalysts are used in the non‐oxidative dehydrogenation of various alkanes (excluding ethane) on large scale,^[^ ^7^, ^8^ ^]^ the high cost of Pt and the toxicity of Cr(VI) compounds limited their widespread application. These shortcomings motivate the researcher to develop cheaper and more environmentally compatible catalysts. Those containing In,^[^ ^9^, ^10^ ^]^ Fe,^[^ ^3^, ^11^ ^]^ Co,^[^ ^4^, ^12^, ^13^, ^14^, ^15^ ^]^ or Ni^[^ ^16^ ^]^ as active components are the most promising candidates.

In recent years, Co‐based catalysts have attracted increasing attention due to their excellent ability to selectively activate C─H bonds in light alkanes.^[^ ^17^, ^18^, ^19^, ^20^ ^]^ In the EDH reaction, isolated,^[^ ^4^, ^12^, ^13^, ^14^, ^15^ ^]^ dimeric Co^2+^ species,^[^ ^4^ ^]^ and/or small CoO*_x_* clusters^[^ ^21^ ^]^ in different siliceous (e.g., HMS, MCM‐41, silicalite‐1, deal‐beta, or deal‐MOR) supports are suggested as the active species. Metallic Co species formed through the reduction of CoO*_x_* nanoparticles during catalyst treatment and/or under EDH conditions are involved in cracking and coke formation reactions.^[^ ^21^ ^]^ Significant drawbacks of all silica‐supported cobalt‐containing catalysts are their low activity and, most importantly, inability to restore their initial performance in a series of dehydrogenation/oxidative regeneration cycles due to the weak CoO*_x_*‐support interactions. These limitations critically undermine the industrial applicability of such catalysts.

Aluminosilicate zeolites, constructed from corner‐sharing [SiO_4_] and [AlO_4_] tetrahedra, provide a robust platform for stabilizing catalytically active metal‐oxide species at the framework [AlO_4_]^−^ sites within well‐defined micropores.^[^ ^22^ ^]^ The combination of spatial confinement and strong electrostatic host–guest interactions enables atomic dispersion of the active species, precise control over their coordination geometry, and exceptional resistance to sintering or reduction under harsh reaction conditions.^[^ ^23^ ^]^ Among these frameworks, the small‐pore chabazite (CHA)‐type SSZ‐13 (pore aperture: 0.38 × 0.38 nm) is particularly notable for its outstanding thermal and hydrothermal stability.^[^ ^24^ ^]^ As a consequence, Cu‐ or Fe‐exchanged SSZ‐13 catalysts have been widely implemented in the selective catalytic reduction (SCR) of NO*_x_* from diesel exhaust under severe and transiently fluctuating operating environments.^[^ ^25^ ^]^ Such an exceptional ability to stabilize metal cations and preserve their structural and electronic integrity under high‐temperature alternating reducing and oxidizing conditions is equally desirable for the EDH reaction.

Taking the above context into consideration, we prepared a series of Co/SSZ‐13 catalysts containing exclusively isolated Co^2+^ ions at different cobalt loadings (xCo; x stands for the weight percentage of Co) and assessed their performance in the EDH reaction. The catalyst with only 0.9 wt% Co showed remarkable ethene productivity at close to equilibrium ethane conversion degrees and unprecedented durability, the ability to restore its initial activity after a larger number of dehydrogenation/oxidative regeneration cycles, in comparison with state‐of‐the‐art catalysts. The precise material synthesis combined with sophisticated multi‐technique analysis enabled us to link unambiguously the high EDH activity to the isolated Co^2+^ ions located in the six‐membered‐ring (6MR) sites of the SSZ‐13 framework. Their counterparts stabilized in the eight‐membered‐ring (8MR) sites are significantly less active and more reducible. Furthermore, we demonstrate strategies to control the relative fraction of these distinct Co^2+^ species. These findings provide a fundamental basis for tailoring cobalt–zeolite architectures toward highly active, selective, and durable catalysts for ethene production.

## Results and Discussion

### Platform of Catalysts and Structure and Location of Cobalt Species

**Figure 1:**
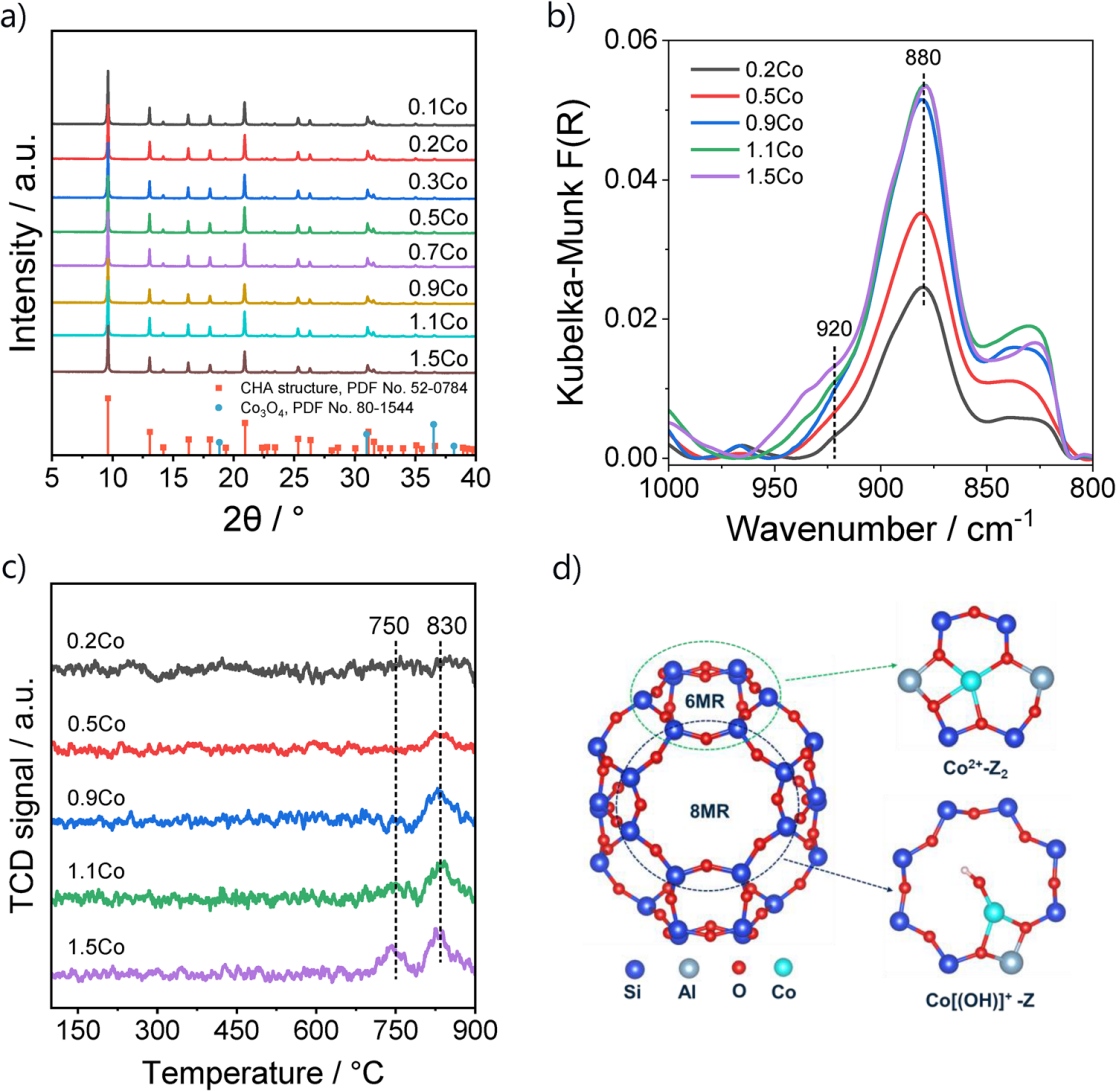
a) Ex situ XRD patterns, b) in situ NH_3_‐DRIFT spectra, and c) H_2_‐TPR profiles of differently loaded Co/SSZ‐13 catalysts. d) DFT‐optimized geometries of the Co^2+^─Z_2_ and [Co(OH)]^+^─Z species located in the 6MR and 8MR windows, shown as representative configurations supported by experimental observations.

Ex situ X‐ray diffraction (XRD) patterns confirmed that all the fresh Co‐containing SSZ‐13 catalysts retained the characteristic SSZ‐13 framework reflections without detectable structural degradation (Figure 1a). No additional reflections attributable to crystalline Co_3_O_4_ phases were observed, indicating the absence of crystalline cobalt oxide aggregates and suggesting a high dispersion of cobalt species. The latter could be confirmed by high‐resolution transmission electron microscopy (HRTEM) analysis of the representative 0.9Co sample with the third highest Co loading (Figure S1a–c), consistent with the XRD results. Energy‐dispersive X‐ray spectroscopy (EDS) mapping (Figure S1d–i) further confirmed the homogeneous spatial distribution of cobalt throughout the SSZ‐13 crystallites, indicative of the high dispersion achieved via ion exchange method. Ultraviolet‐visible (UV–vis) spectra further support this conclusion (Figure S2). The spectra of all Co/SSZ‐13 samples do not have absorption bands near 740 nm, typically assigned to the d–d transitions of cobalt oxide, thereby excluding the presence of CoO*_x_* clusters detectable by this technique.^[^ ^26^ ^]^ Instead, the spectra are dominated by features that are characteristic of the tetrahedrally and/or octahedrally coordinated Co^2+^ ions associated with the zeolite framework. Ex situ X‐ray photoelectron spectroscopy (XPS) of representative 0.5Co, 0.9Co, and 1.5Co samples (Figure S3) consistently revealed only Co^2+^ species (Co 2p_3/2_ at 782.2 eV with a shake‐up satellite at 788.1 eV),^[^ ^27^ ^]^ demonstrating that framework‐stabilized Co^2+^ cations are the exclusive cobalt species across the entire loading range. In samples with Co loadings ≤0.3 wt%, the Co 2p signal was below the detection limit. These isolated Co^2+^ ions were introduced into SSZ‐13 by replacing Brønsted protons at ion‐exchange sites, thereby generating new Lewis acid sites (Figure S4).

In divalent metal‐exchanged zeolites, the strong interaction between cations and framework oxygen perturbs the asymmetric T–O–T vibrations, giving rise to distinct infrared (IR) bands at about 950–850 cm^−1^ that depend on the valence, size, and location of the cations.^[^ ^28^, ^29^ ^]^ These perturbations are more clearly resolved when NH_3_ is introduced as a probe molecule coordinated to the cations, resulting in well‐defined T–O–T bands in differential spectra.^[^ ^30^ ^]^ Accordingly, diffuse reflectance infrared Fourier transform spectroscopy with NH_3_ (NH_3_‐DRIFTS) was employed to characterize the nature of Co^2+^ ions in Co/SSZ‐13. Two distinct bands are seen in the spectra at about 920 and 880 cm^−1^ (Figures 1b and S5), indicating the coexistence of two types of Co^2+^ ions occupying different ion‐exchange sites in the zeolite framework.

Given the close similarity in the charge and the ionic radii of Co^2+^ and Cu^2+^, the coordination motifs of Co^2+^ in the SSZ‐13 framework are expected to be in parallel to those of Cu^2+^ in the same zeolite,^[^ ^31^, ^32^ ^]^ i.e. Co^2+^ balanced by two framework negative charges (Co^2+^─Z_2_, with Z representing a charged zeolite framework site) and [Co(OH)]^+^ (Co hydroxyl) stabilized by a single framework charge ([Co(OH)]^+^─Z). The presence of the latter species in our catalysts is supported by the presence of an IR band at 3670 cm^−1^ in the *ν*(OH) region (Figure S6) caused by NH_3_ adsorbed on the [Co(OH)]^+^ species.^[^ ^33^ ^]^ Combining operando XAS, FTIR, kinetic, and DFT studies reported previously,^[^ ^33^, ^34^ ^]^ together with the high Si/Al ratio of 21 in our SSZ‐13, Co^2+^ species require two nearby framework charges and therefore should be preferentially stabilized in Al pairs within the six‐membered rings (6MRs) as Co^2+^─Z_2_. Contrarily, the [Co(OH)]^+^ species requiring a single framework charge are located in the eight‐membered rings (8MRs) as [Co(OH)]^+^─Z.

Analysis of the NH_3_‐DRIFT spectra (Figure 1b) reveals two distinct vibrational perturbations of the zeolite framework, characterized by the bands at about 920 and 880 cm^−1^. The intensity of the 880 cm^−1^ band increases progressively with cobalt loading up to 0.9 wt%, while that of the 920 cm^−1^ band shows only a slight increase over the same loading range. Beyond 0.9 wt% loading, the 880 cm^−1^ band reaches a plateau, whereas the 920 cm^−1^ feature becomes significantly more pronounced. From a thermodynamic perspective, the more stable 6MR sites are preferentially occupied by Co^2+^ ions, and only upon their saturation, additional Co^2+^ ions incorporate into the less stable 8MR sites.^[^ ^35^ ^]^ Accordingly, the 880 cm^−1^ band can be attributed to the framework perturbations induced by the Co^2+^─Z_2_ species, while the 920 cm^−1^ band corresponds to the [Co(OH)]^+^─Z species. In addition, a shoulder band at about 828 cm^−1^ is also observed, particularly at higher Co loadings. Its intensity increases in parallel with that of the 880 cm^−1^ band, suggesting that it reflects an additional perturbation of the framework vibrations associated with the increasing content of the Co^2+^─Z_2_ species.

H_2_‐TPR measurements were performed to probe the reducibility of Co/SSZ‐13 catalysts, a property closely related to both dehydrogenation activity and durability of different catalysts.^[^ ^36^ ^]^ No obvious consumption of H_2_ over all catalysts could be seen below 700 °C (Figure 1c). Thus, the presence of even small amounts of cobalt oxides and/or CoO*_x_* (sub)nanoclusters can be excluded. Such species are typically reduced in the temperature ranges of 100–400 and 400–600 °C, respectively.^[^ ^37^ ^]^ The H_2_‐TPR profiles of the 0.2Co, 0.5Co, and 0.9Co catalysts are characterized by only a weak peak of H_2_ consumption with the maximal rate at about 830 °C. In contrast, the H_2_‐TPR profile of the 1.1Co and 1.5Co catalysts is characterized by an additional H_2_ consumption with the maximal rate at about 750 °C. Notably, the intensity of the 830 °C peak increases progressively with Co loading up to 0.9 wt% but remains essentially constant at higher loadings. In light of the NH_3_‐DRIFTS results, the high‐temperature consumption of H_2_ can be assigned to the reduction of the Co^2+^─Z_2_ species, whereas the 750°C feature should originate from the reduction of the [Co(OH)]^+^─Z species. These observations not only demonstrate the remarkable thermal stability of cobalt species strongly anchored within the SSZ‐13 framework but also provide compelling evidence for the coexistence of two distinct types of isolated Co^2+^ ions, whose relative abundance evolves systematically with Co loading.

Overall, the application of the complementary techniques enabled us to identify two types of isolated Co^2+^ ions located in the 6MR and 8MR sites of SSZ‐13 (Figure 1d). Their relative fraction can be controlled by the loading of Co. Further structural details obtained from in situ XAS analysis are thoroughly discussed in a separate section after the discussion of the results of the catalytic tests in the below section. The purpose of such tests was to answer whether and how the molecular structure of the differently located Co^2+^ ions affects their activity and selectivity in the EDH reaction.

### Catalytic Performance and Active‐Site Identification in Co/SSZ‐13

**Figure 2:**
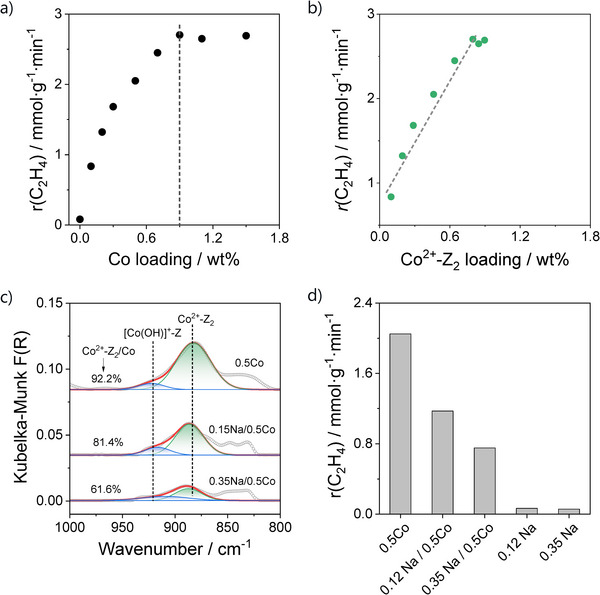
The rate of ethene formation over differently loaded Co/SSZ‐13 versus a) Co loading or b) Co^2+^─Z_2_ content determined from Figure S5. c) The NH_3_‐DRIFT spectra of the 0.5Co, 0.12Na/0.5Co, and 0.35Na/0.5Co catalysts. d) The rate of ethene formation over the 0.5Co, 0.12Na/0.5Co, 0.35Na/0.5Co, 0.12Na, and 0.35Na catalysts. Reaction conditions for (a) and (d): *T* = 600 °C, C_2_H_6_:N_2_ = 0.2:0.8, WHSV(C_2_H_6_) = 48.2 h^−1^.

The rate of ethene formation over differently loaded Co/SSZ‐13 catalysts is shown in Figure 2a. As the bare SSZ‐13 support showed negligible activity, the formation of ethene over the Co‐containing materials should be related to the presence of isolated Co^2+^ species. Ethene formation rate increases markedly with increasing Co content, reaching a maximum of 2.7 mmol g^−1^ min^−1^ at 0.9 wt% Co. As no further increase in the rate was achieved when the loading increased beyond this point, it is likely that the added cobalt forms other isolated Co^2+^ ions that are significantly less active, rather than generating the active sites. To explain the activity‐Co‐content relationship in Figure 2a, we refer to the results of the catalyst characterization shown in Figure 1b,c. They suggest that the Co^2+^─Z_2_ species are preferentially formed in the 6MR sites at a Co loading up to 0.9 wt%. When the loading increases further, [Co(OH)]^+^─Z species stabilized in the 8MR sites are mainly formed, while the concentration of the Co^2+^─Z_2_ species is not affected. Based on the effects of Co loading on the concentration of the latter species and the rate of ethene formation, we put forward that the Co^2+^─Z_2_ species should be responsible for the high activity of the Co/SSZ‐13 catalysts in the EDH reaction. The [Co(OH)]^+^─Z species must be significantly less active. This statement is supported by the results of the semi‐quantitative Gaussian fitting of the NH_3_‐DRIFTS spectra, which we performed to estimate the Co^2+^─Z_2_ content in the Co/SSZ‐13 catalysts (Table S1). A strong positive correlation between the ethene formation rate and this content was established (Figure 2b), directly linking this type of Co^2+^ site to the catalyst performance.

To provide direct evidence for the key role of the Co^2+^─Z_2_ sites in the EDH reaction, we prepared a series of Na/Co/SSZ‐13 catalysts containing different amounts of Na but the same Co loading of 0.5 wt%, which is suitable to preferentially form isolated Co^2+^ species in the 6MR window in the absence of Na^+^ (Figure 2c). It is important to note that the Na‐containing samples were obtained by deliberately adjusting the NH_4_NO_3_ concentration during the ammonium‐exchange step preceding cobalt ion exchange, thereby retaining controlled amounts of Na^+^ ions in the zeolite. During ammonium exchange, Na^+^ cations located in the more accessible 8MR sites are readily replaced by NH_4_^+^, whereas those in the sterically constrained 6MR windows are more difficult to displace and typically require multiple exchange cycles for nearly complete removal.^[^ ^38^ ^]^ As a result, residual Na^+^ ions in the Na/0.5Co/SSZ‐13 samples predominantly occupy the 6MR positions, restricting the availability of these sites to Co^2+^ ions. Consequently, Co^2+^ ions are forced to enter into the 8MR windows, forming the [Co(OH)]^+^─Z species. In agreement with the above discussion, our NH_3_‐DRIFTS analysis demonstrated that the intensity of the 880 cm^−1^ band characteristic of Co^2+^─Z_2_ decreased, while the relative contribution of the 920 cm^−1^ band typical for [Co(OH)]^+^─Z increased when the amount of Na in the Na/Co/SSZ‐13 catalysts increased (Figure 2c). Consistent with the reduced fraction of the Co^2+^─Z_2_ species, the results of EDH tests with these catalysts revealed that the ethene formation rate decreases significantly with increasing Na content (Figure 2d).

Taken together, these results provide compelling experimental evidence that the Co^2+^─Z_2_ sites are the dominant active centers in the EDH reaction over Co/SSZ‐13, whereas the [Co(OH)]^+^─Z species contribute negligibly to the activity. Insights into the local structure of the Co^2+^─Z_2_ sites were derived from the below XAS analysis and were used to rationalize the origin of catalytic performance in the EDH reaction through DFT calculations.

### In Situ XAS Investigation of Co/SSZ‐13 Under Reductive Conditions Simulating EDH

**Figure 3:**
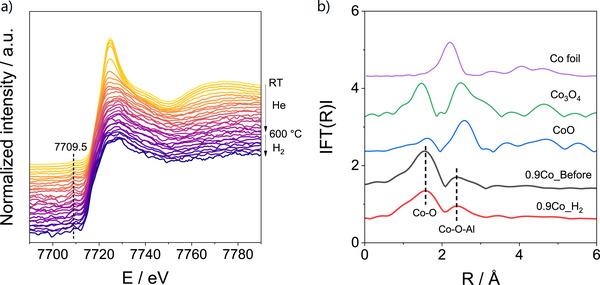
a) In situ XANES spectra of 0.9Co at the Co K‐edge during heating in He to 600°C followed by reduction in 50 vol% H_2_/He. b) FT EXAFS spectra (not corrected for the phase shift) of 0.9Co at room temperature and after H_2_ treatment at 600°C as well as reference samples (Co foil, CoO, and Co_3_O_4_).

To assess the structural and electronic information about cobalt species during EDH, in situ XAS tests were conducted using the most active 0.9Co catalyst. In these experiments, hydrogen was used as a reducing probe molecule to mimic the reducing character of the EDH environment while avoiding complications from coke formation during the EDH process. The catalyst was first heated to 600 °C in flowing He, followed by exposure to a flow of 50 vol% H_2_ in He for 30 min (Figure 3a).

The XANES spectrum of 0.9Co at room temperature is characterized by the pre‐edge energy at 7709.5 eV, indicating the oxidation state of cobalt of 2+ (Figure 3a).^[^ ^17^, ^18^ ^]^ This observation is consistent with the ex situ XPS results. During the heating process in He, the intensity of the white line decreases, while the position of the cobalt pre‐edge stays at 7709.5 eV. This suggests some changes in the local coordination of Co^2+^ without changing in the cobalt oxidation state. Similar spectral shapes and trends were observed in the XANES spectra of Cu/SSZ‐13 during heating in 10 vol%O_2_/He, which were attributed to stepwise dehydration of Cu^2+^ species.^[^ ^39^ ^]^ After switching to a flow of H_2_ at 600 °C, no significant changes in the XANES spectra were observed, indicating that the Co state remained stable during the reduction treatment. This result is consistent with the H_2_‐TPR results, which showed that none of the cobalt species in the 0.9Co catalyst undergo reduction at 600 °C (Figure 1c).

According to the fit of the EXAFS spectra of 0.9Co at room temperature (0.9Co_Before) and after H_2_ treatment (0.9Co_H_2_) at 600 °C (Figures 3b, S7 and Table S2), the Co^2+^ species in the as‐prepared 0.9Co_Before sample should have two distinct Co─O paths with bond lengths of 2.00 and 2.16 Å, along with a Co─O─Al path at 2.81 Å. The longer Co─O distance (2.16 Å) is attributed to the coordination with hydroxyl ligands or adsorbed water molecules,^[^ ^6^, ^7^ ^]^ whereas the shorter Co─O bond corresponds to the interaction of Co^2+^ with the framework lattice oxygen, confirming that cobalt exists as isolated Co^2+^ ions anchored to the zeolite lattice. After heating to 600°C and H_2_ treatment at this temperature, the longer Co─O contribution disappeared, consistent with the removal of coordinated hydroxyl/water ligands, while the shorter Co─O bond retained a coordination number (CN) of about 3.5. The second‐shell Co─O─Al interaction also remained essentially unchanged (CN of about 2.1), demonstrating that the isolated Co^2+^ ions preserved their framework‐associated geometry under reducing conditions at 600°C.

Overall, the in situ XAS results demonstrate that isolated Co^2+^ ions predominantly present in the 0.9Co catalyst are structurally and electronically stable under a reducing environment simulating EDH.

### DFT Insights into Reaction Pathways over Different Co Sites in Co/SSZ‐13

**Figure 4:**
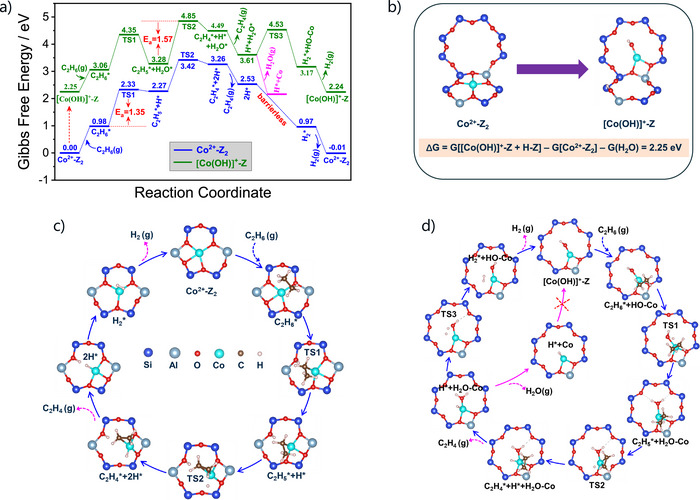
The calculated free energy diagram in the course of the EDH reaction over the Co^2+^–Z_2_ and [Co(OH)]^+^–Z sites with the corresponding structures along the reaction paths.

DFT calculations were performed to elucidate the molecular‐level reaction pathways in the course of the EDH reaction over the Co^2+^–Z_2_ and [Co(OH)]^+^–Z sites. The Gibbs free energy profiles under the experimental reaction conditions are shown in Figure 4a, and the calculated Bader charge analysis is provided in Figure S8. Importantly, the transformation from Co^2+^–Z_2_ to [Co(OH)]^+^–Z in the 8MR environment entails an intrinsic free‐energy penalty of 2.25 eV (Figure 4b).

For the Co^2+^–Z_2_ site (Figure 4c), C_2_H_6_ activation begins with the cleavage of the first C─H bond, generating *H^δ+^ and *C_2_H_5_^δ−^ species with an activation barrier of 1.35 eV. The *C_2_H_5_^δ−^ intermediate then undergoes another C─H bond cleavage to form *H^δ−^ and *C_2_H_4_ with an activation barrier of 1.15 eV. The formed C_2_H_4_ easily desorbs (Δ*G* = −0.73 eV). The *H^δ+^ and *H^δ−^ species react with each other barrierless to form *H_2_, which desorbs readily, regenerating the active Co^2+^─Z_2_ site and thus completing the catalytic cycle.

In contrast, the [Co(OH)]^+^─Z site features a hydroxyl ligand that enables a direct interaction between H atom in C_2_H_6_ and the Co─OH group (Figure 4d). The OH group of the [Co(OH)]^+^─Z site abstracts the H in C_2_H_6_, leading to the first C─H bond cleavage, producing *C_2_H_5_^δ−^ and *H_2_O with an activation barrier of 1.29 eV. However, the subsequent dehydrogenation of *C_2_H_5_^δ−^ to *C_2_H_4_ and *H^δ−^ requires a barrier of 1.57 eV, which is higher than that at the Co^2+^–Z_2_ site, underscoring the strong influence of the local coordination environment on the reaction energetics. Ethene desorption remains thermodynamically favorable (Δ*G* = −0.88 eV). Then, the *H^δ−^ species can subtract H^+^ from *H_2_O to yield *H_2_ and regenerate the [Co(OH)]^+^─Z site. This step involves an additional 0.92 eV barrier. However, *H_2_O desorption from the Co site is highly exergonic (Δ*G* = −1.45 eV), indicating water removal is thermodynamically favored over [Co(OH)]^+^─Z active‐site regeneration, and this thereby potentially interrupts the catalytic cycle.

Based on the overall free‐energy landscapes in Figure 4a, the rate‐determining step (RDS) in the EDH reaction over the Co^2+^─Z_2_ and [Co(OH)]^+^─Z sites is the cleavage of first and second CH bonds in C_2_H_6_, respectively. However, the former step needs lower barrier than the latter one, i.e., 1.35 eV versus 1.57 eV, suggesting the higher reactivity of the 6MR Co^2+^─Z_2_ site. This conclusion is further supported by the density of states (DOS) analysis (Figure S9), which shows that the Co 3d‐band center in the 6MR framework lies closer to the Fermi level, facilitating electron donation from Co 3d to the σ* antibonding orbital of the C─H bond and thereby promoting C─H bond activation. Additional calculations show that moderate variations in temperature (773–873 K) (Figure S10) or pressure (0.5–2 atm) (Figure S11) introduce only negligible changes (≤0.02 eV) to the activation barriers, and the reactivity order (Co^2+^─Z_2_ > [Co(OH)]^+^─Z) remains unchanged. This demonstrates that the mechanistic conclusions are valid for typical EDH conditions.

### Industrial Relevance and Benchmarking of Co/SSZ‐13

**Figure 5:**
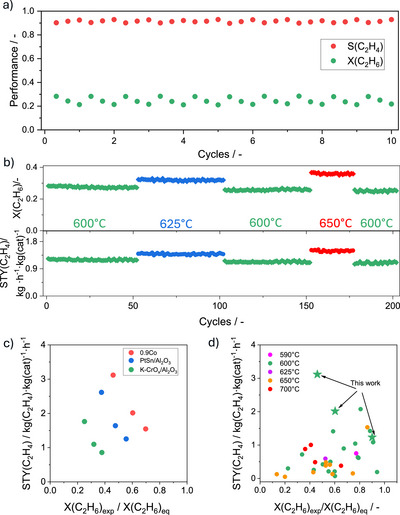
a) Ethane conversion (X(C_2_H_6_)) and ethene selectivity (S(C_2_H_4_)) over 0.9Co in the first 10 EDH/regeneration cycles at 600°C. b) On‐stream profiles of ethane conversion (X(C_2_H_6_)) and space‐time yield of ethene formation (STY(C_2_H_4_)) over 0.9Co in a series of 200 EDH/regeneration cycles at 600°C (green), 625°C (blue), and 650°C (red). Reaction conditions: *T* = 600–650°C, C_2_H_6_:N_2_ = 0.2:0.8, WHSV(C_2_H_6_) = 5.1 h^−1^. c) STY(C_2_H_4_) determined over Pt‐Sn/Al_2_O_3_, K‐CrO*_x_*/Al_2_O_3_, and 0.9Co at different X(C_2_H_6_)_exp_/X(C_2_H_4_)_eq_. d) STY(C_2_H_4_) determined over 0.9Co and previously tested catalysts (Table S3) at different temperatures versus X(C_2_H_6_)_exp_/X(C_2_H_4_)_eq_. Reaction conditions for (c): *T* = 600°C, C_2_H_6_:N_2_ = 0.2:0.8, WHSV(C_2_H_6_) = 5.1–24.1 h^−1^.

The ability of an alkane‐dehydrogenation catalyst to recover its initial performance after oxidative regeneration is a critical requirement for commercial application. Although the conversion of ethane over our best‐performing 0.9Co catalyst decreased within one EDH cycle, it could reach its initial value after oxidative catalyst regeneration (Figure 5a). Thus, we tested this catalyst in a series of 200 consecutive dehydrogenation/regeneration cycles conducted at 600, 625, and 650°C using a feed containing 20 vol% ethane (Figure 5b). The total experiment time was about 150 h. The selectivity to ethene also remained stable and was above 85% (Figure S12). The decrease in ethane conversion is caused by the formation of coke from ethene on acidic sites. This undesired product was effectively removed during the oxidative regeneration step. The heat produced through coke combustion and stored by the catalyst can be used to power the highly endothermic EDH reaction as in the Catofin propane dehydrogenation (PDH) process. In this process, one PDH cycle lasts only 7–15 min on stream, followed by oxidative regeneration, resulting in a total cycle time of 15–30 min.^[^ ^36^, ^40^ ^]^ Therefore, the low on‐stream performance of the developed Co/SSZ‐13 catalyst within one EDH cycle does not inherently undermine its practical relevance.

Post‐reaction HRTEM imaging and EDS elemental mapping of the catalyst after 200 cycles confirmed that Co^2+^ remained highly dispersed, no agglomerated cobalt species could be observed (Figure S13). Our additional XAS analysis further confirms that the structure of the isolated Co^2+^ species did not change after 200 EDH/regeneration cycles proving their high stability against sintering (Figures S14 and S15).

We also tested the 0.9Co catalyst in parallel with analogues of commercial Pt─Sn/Al_2_O_3_ and K─CrO*_x_*/Al_2_O_3_ catalysts, which have, however, been developed for the non‐oxidative dehydrogenation of propane to propene, at 600°C using a feed with 20 vol% C_2_H_6_ in N_2_ at different weight hourly space velocities. As expected, the space‐time yield of ethene formation (STY(C_2_H_4_)) decreased with increasing X(C_2_H_6_)_exp_/X(C_2_H_6_)_eq_ (X(C_2_H_6_)_exp_ and X(C_2_H_6_)_eq_ stand for the experimental and equilibrium ethane conversion, respectively.) (Figure 5c), reflecting the thermodynamic limitation as equilibrium is approached. At 46%, 48%, and 38% equilibrium conversion degrees, the STY(C_2_H_4_) values of 0.9Co, Pt─Sn/Al_2_O_3_, and K─CrO*_x_*/Al_2_O_3_ were 3.1, 1.7, and 0.85 kg(C_2_H_4_) kg_cat_^−1^ h^−1^, respectively, demonstrating that 0.9Co outperforms the commercial benchmarks by factors of about 2 and 4.

The performance of the 0.9Co catalyst was further benchmarked against state‐of‐the‐art EDH catalysts in terms of STY(C_2_H_4_). To ensure a fair comparison of the catalysts tested under different reaction conditions, it is important to consider the corresponding equilibrium conversion values, since catalyst activity decreases as equilibrium is approached (Figure 5c). Thus, we plotted the STY(C_2_H_4_) values versus X(C_2_H_6_)_exp_/X(C_2_H_6_)_eq_ (Figure 5d). The 0.9Co catalyst achieved the STY(C_2_H_4_) values of 3.1, 2.0, and 1.2 kg(C_2_H_4_) kg_cat_^−1^ h^−1^ at 46%, 60%, and 90% equilibrium ethane conversion at 600°C, respectively. These values surpass those of most previously reported EDH catalysts tested even at higher temperatures. A recently developed CoO*_x_*/zeolite beta catalyst showed a slightly higher productivity.^[^ ^4^ ^]^ This catalyst also predominantly possesses isolated Co^2+^ species, highlighting the importance of the use of zeolite support to prepare highly active catalysts. These Co^2+^ species are stabilized exclusively by silanol defects formed after dealumination of zeolite beta. Contrarily, the Co^2+^ ions in our Co/SSZ‐13 system preferentially occupy paired framework‐Al sites located in the 6MR windows, forming structurally well‐defined isolated Co^2+^─Z_2_ species. The latter species are highly stable against reaction‐induced restructuring even at 650°C, thus ensuring the catalyst operates durably. The authors from Ref. [4] did not report on the durability of the isolated Co^2+^ species in the CoO*_x_*/zeolite beta catalysts in the EDH reaction at temperatures above 550°C.

The high ethylene productivity at close‐to‐equilibrium ethane conversion, coupled with the unexpectedly high durability (Figure 5b), establishes the 0.9Co catalyst as a promising candidate for industrial ethane dehydrogenation. This auspicious catalyst performance together with the knowledge obtained about the type of active sites and how they are purposefully prepared, could lead to the development of even more efficient catalysts based not only on cobalt but also on other metals, additionally utilizing suitable promoters.

## Conclusion

In summary, Co/SSZ‐13 catalysts with isolated Co^2+^‐containing species are identified as the first highly durable EDH system outperforming most of previously developed EDHcatalysts in terms of ethene productivity at industrially relevant degrees of ethane conversion and ethene selectivity. The optimized 0.9Co catalyst fully recovers its initial performance over 200 reaction–regeneration cycles at 600–650 °C lasting in total for about 150 h. Comprehensive spectroscopic, microscopic, and temperature‐programmed analyses reveal that the catalytically active species are located in the 6MR windows (Co^2+^─Z_2_ species, with Z representing a charged zeolite framework site), while Co^2+^‐containing species located in the 8MR windows ([Co(OH)]^+^─Z species) play a minor role. The former sites are preferentially formed at low Co loadings until the free 6MR sites are close to saturation. In situ XAS measurements confirm that the Co^2+^─Z_2_ sites are structurally and electronically stable under reductive conditions. DFT calculations revealed the origins of the different activity of the Co^2+^‐containing species located in the 6MR and 8MR windows. The Co^2+^─Z_2_ sites possess a lower activation barrier for the rate‐determining C─H cleavage, a favorable electronic configuration for C─H bond activation, and an uninterrupted catalytic cycle, in stark contrast to the [Co(OH)]^+^─Z sites, where facile water desorption disrupts active‐site regeneration. Thus, this work not only delivers an active and durable EDH catalytic system but also establishes the atomic‐level design principles required to bring next‐generation Co‐zeolite catalysts to commercial applications.

## Conflict of Interests

The authors declare no conflict of interest.

## Supporting information



Supporting Information

## Data Availability

The data that support the findings of this study are available from the corresponding author upon reasonable request.
