# Supplementary material for: SSZ‐13 Zeolite with Isolated Co2+ Sites as an Efficient and Durable Catalyst System for Non‐Oxidative Ethane Dehydrogenation
Source: Angew Chem Int Ed Engl. 2026 Jan 7;65(7):e19600. doi: 10.1002/anie.202519600 (PMC12887641; doi:10.1002/anie.202519600)
Supplement: Supplementary file 1 — Supporting Information [file ANIE-65-e19600-s001.pdf]

Supporting Information

SSZ-13 Zeolite with Isolated Co^{2+} Sites as an Efficient and Durable Catalyst System for Non-Oxidative Ethane Dehydrogenation

Qiyang Zhang,[b] Tao Zhang,*[a,b] Bing Liu,*[c] Elizaveta Fedorova,[b] Dmitry E. Doronkin,[d] and Evgenii V. Kondratenko*[b]

[a] Prof. Dr. T. Zhang
School of Chemistry and Life Resources
Renmin University of China
Beijing 100872, P. R. China
E-mail: zhangt@ruc.edu.cn (TZ)

[b] Dr. Q. Zhang, Prof. Dr. T. Zhang, Dr. E. Fedorova, Prof. Dr. E. V. Kondratenko
Department of Advanced Methods for Applied Catalysis
Leibniz-Institut für Katalyse e.V. (LIKAT)
Albert-Einstein-Str. 29a, 18059 Rostock (Germany)
E-mail: Evgenii.Kondratenko@catalysis.de (EVK)

[c] Prof. Dr. Bing Liu
Department of Chemical Engineering, School of Chemical and Material Engineering
Jiangnan University
Wuxi 214122, P. R. China
E-mail: liubing@jiangnan.edu.cn

[d] Dr. D. M. Doronkin
Institute for Chemical Technology and Polymer Chemistry, and Institute of Catalysis Research and Technology
Karlsruhe Institute of Technology
Kaiserstr. 12, D-76131, Karlsruhe (Germany)

SUPPORTING INFORMATION

Table of Contents

Experimental Section…………………………………………………………………………… ………………...…………….3

Supporting Figures………7

Supporting Tables…………..…………………………………………………………………………………………….22

Supporting References…………...…………..………………………………………………………………………..28

Experimental Section

Catalyst synthesis

Na/SSZ-13 was synthesized in-house following a previously reported hydrothermal procedure.[1] The as-synthesized material was converted into the NH_4/SSZ-13 form by ammonium exchange with an excess of 1 M NH_4NO_3 at 80 °C for 12 h, repeated twice to ensure complete exchange. According to inductively coupled plasma optical emission spectroscopy (ICP–OES) analysis, the calcined NH_4/SSZ-13 material has a Si/Al ratio of about 21. Co/SSZ-13 samples with different amounts of Co were prepared using the solution ion exchange method. Specifically, eight $Co(NO_3)_2$ solutions were prepared by dissolving an appropriate amount of $Co(NO_3)_2$ in 100 mL of deionized water, and the pH value was adjusted to about 3.5 by adding diluted HNO_3. One gram of NH_4/SSZ-13 was then added to each solution, and ion exchange was conducted at 80 °C for 12 h. The solids were recovered by centrifugation, thoroughly washed with deionized water, dried at 100 °C for 12 h, and calcined at 600 °C for 8 h. ICP–OES analysis determined the Co loadings of about 0.1, 0.2, 0.3, 0.5, 0.7, 0.9, 1.1, and 1.5 wt%. The prepared Co/SSZ-13 materials are abbreviated as *x*Co, where *x* represents the Co loading (wt%).

Additionally, two comparative samples with an identical cobalt loading of 0.5 wt% but different sodium contents were prepared by varying the concentration of the ammonium nitrate solution during the ammonium exchange step. The synthesis procedure followed that described above, except that 0.01 M and 0.001 M NH_4NO_3 solutions were used for the ammonium exchange. To maintain the same final cobalt loading, the duration of the subsequent cobalt ion-exchange step was adjusted accordingly. ICP analysis revealed sodium content of about 0.12 wt% and 0.35 wt%, respectively, while the cobalt content was about 0.5 wt% in both cases. These samples are hereafter referred to as 0.12Na/0.5Co and 0.35Na/0.5Co.

For benchmarking purpose, two industrially relevant catalysts were synthesized as reference systems. An analogue of commercial K–CrO_x/Al_2O_3 was synthesized according to the method described in the patent from Vladimir Fridman.[2] Briefly, desired amounts of $Cr(NO_3)_3{\cdot}9H_2O$ and KOH were separately dissolved in water. The two solutions were then mixed. Afterwards, a commercial Al_2O_3 support (Saint-Gobain) was added to the above solution. The catalyst was collected after drying and calcination at 760 °C for 4 h. The amount of Cr_2O_3 and K_2O in the resulting catalyst was 19.7 wt% and 0.93 wt%, respectively.

Similarly, an analogue of the industrial Pt–Sn/Al_2O_3 catalyst (Pt: 0.5 wt.% and Sn:1.5 wt.%) was prepared by impregnation.[3] First, a solution containing metal precursors was prepared by dissolving 0.628 g of H_2PtCl_6 (8 wt%H_2Cl_6Pt; Aldrich) and 0.145 g of $SnCl_2{\cdot}2H_2O$ (99.99%, Sigma-Aldrich) in 30 ml of 99.9% ethanol. Hereafter, five grams of a commercial Al_2O_3 support (Saint-Gobain) were impregnated with the solution. The resulting sample was dried in an oven at 100 °C for 12 h and then calcined in air at 560 °C for 3 h.

Catalyst characterization

The elemental composition of SSZ-13 and Co/SSZ-13 samples was determined by inductively coupled plasma optical emission spectroscopy (ICP–OES) using an Agilent 720 instrument.

Brunauer–Emmett–Teller (BET) surface areas were measured on a Micromeritics ASAP 2020 analyzer. Prior to analysis, all samples were degassed in vacuum at 300 °C for 4 h.

Powder X-ray diffraction (XRD) patterns were recorded on a Rigaku SmartLab 3 kW diffractometer equipped with automatic divergence slits, using Cu $K\alpha_1/\alpha_2$ radiation (λ = 0.15406/0.15444 nm, 40 kV, 40 mA).

High-resolution transmission electron microscopy (HRTEM) images and energy-dispersive X-ray spectroscopy (EDS) elemental mapping were performed on an FEI Talos 200X microscope operated at 200 kV to examine the microstructure and the elemental distribution of the fresh and spent catalysts.

Ultraviolet-visible (UV-vis) diffuse reflectance spectra of all catalysts were recorded using an Avantes AvaSpec-2048-USB2-RM spectrometer equipped with a high-temperature reflection UV–vis probe, an AvaLight-DH-S-BAL deuterium–halogen light source, and a CCD array detector. The probe, consisting of six illumination fibers and one collection fiber, was inserted through the furnace to face the wall of the quartz tubular reactor at the position of the

catalyst bed (200 mg). The UV-vis spectra were collected at room temperature in the wavelength range of 200–1100 nm. Barium sulfate (99.998%, Aldrich) was used as the reflectance standard.

X-ray photoelectron spectroscopy (XPS) measurements were performed on a Thermo Fisher Scientific ESCALAB 220iXL spectrometer using monochromatic Al Kα radiation (E = 1486.6 eV). Each catalyst sample was mounted on a stainless-steel holder using conductive double-sided carbon adhesive tape. Electron binding energies were measured with charge compensation provided by a flood electron source and calibrated against the C 1s peak of adventitious carbon at 284.8 eV.

NH_3 temperature-programmed desorption (NH_3-TPD) tests were carried out on a TP-5080 adsorption analyzer. Approximately 50 mg of sample was pretreated in He flow (30 mL min^{-1}) at 550 °C for 1 h, followed by cooling to ambient temperature in the same flow. Using a feed with 1 vol% NH_3/Ar, NH_3 adsorption was performed at 100 °C for 1 h followed by feeding He for 1 h to remove physically adsorbed NH_3. Thermal conductivity detector (TCD) was used to detect the desorption of NH_3 while the sample was heated from ambient temperature to 700 °C at a heating rate of 10 °C min^{-1}.

In situ diffuse reflectance infrared Fourier transform spectroscopy (DRIFTS) measurements were performed on a Bruker INVENIO S spectrometer equipped with a liquid-nitrogen-cooled mercury cadmium telluride (MCT) detector. For each experiment, about 0.06 g of catalyst was uniformly packed into a high-temperature reaction cell. Prior to data acquisition, the catalyst was calcined in situ at 600 °C for 1 h in a flow of air. The oxidized sample was then cooled to the target reaction temperature in the same flow, and a background spectrum was recorded. Subsequently, a gas mixture containing 1 vol% NH_3 in Ar was introduced at a total flow rate of 20 mL min^{-1} and maintained throughout the measurement. The DRIFTS spectra were collected at a resolution of 4 cm^{-1}, averaging 100 scans per spectrum, to enable real-time monitoring of surface species and reaction intermediates.

H_2 temperature-programmed reduction (H_2-TPR) tests were performed on a Micromeritics AutoChem II 2920 analyzer. Approximately 50 mg of catalyst was loaded into a U-shaped quartz tube and pretreated in a He flow (50 mL min^{-1}) by heating to 300 °C at 10 °C min^{-1} and holding for 30 min to remove adsorbed species. After cooling to 30 °C in He, the sample was exposed to a flow (50 mL min^{-1}) of 10 vol% H_2/Ar while heating from 30 to 900 °C at a heating rate of 10 °C min^{-1}. H_2 consumption was monitored by a thermal conductivity detector (TCD).

X-ray absorption spectra at the Co K absorption edge were recorded at the P65 beamline of PETRA III synchrotron radiation source (DESY, Hamburg) in transmission mode. Higher harmonics were rejected by a pair of Si plane mirrors installed in front of the monochromator. The energy of the X-ray photons was further selected by a Si(111) double-crystal monochromator and the beam size was set by means of slits to 0.4 (vertical) x 2.0 (horizontal) mm^2. The spectra were normalized and the extended X-ray absorption fine structure spectra (EXAFS) background was subtracted using the ATHENA program from the IFEFIT software package.[4] The k^2-weighted EXAFS functions were Fourier transformed (FT) in the k range of 3.0-9.0$Å^{-1}$ and multiplied by a Hanning window with sill size of 1 $Å^{-1}$. The FT EXAFS spectra were not corrected for the phase shift. For in situ XAS measurements, the 0.9Co catalyst with a sieve fraction of 100-200 μm was loaded in an in situ micro-reactor (quartz capillary, 1.5mm diameter, 0.02mm wall thickness). The sample was heated to 600 °C with a heating rate of 10 K·min^{-1} in He (20 mL·min^{-1} flow rate), followed by feeding a flow of 50 vol% H_2 in He for 30 min. Before recording the spectra, the sample was cooled down in 50 vol% H_2 and kept at room temperature for 10 min.

Catalytic tests

Catalytic tests were initially performed at 600 °C and 1 bar in an in-house-designed setup comprising 15 continuous-flow fixed-bed quartz reactors. The initial rate of ethene formation was determined at an ethane conversion below 10% after the first 300 s on stream. Typically, catalysts (10 mg, particle size 315–710 μm) were heated to 600 °C in N_2 at a heating rate of 10 °C min^{-1}, followed by exposure to a flow (20 mL·min^{-1}) of 20 vol% C_2H_6/N_2.

The durability of the 0.9Co catalyst was evaluated in a series of 200 EDH/regeneration cycles at 600, 625, and 650 °C using a flow (40 mL·min^{-1}) of 20 vol% C_2H_6/N_2 and 125 mg of catalyst. Each EDH cycle lasted 15 min, followed by regeneration in air for 10 min at the same temperature. A 10 min N_2 purge was applied between the

EDH and regeneration steps. Before the first EDH cycle, the catalyst was heated to 600 °C in N_2, then switched to the reaction gas flow.

To obtain different ethane conversion degrees at 600 °C, 0.9Co, Pt-Sn/Al_2O_3 and K-CrO_x/Al_2O_3 catalysts were tested at ethane weigh hourly space velocities (WHSV(C_2H_6)) ranging from 5.1 to 24.1 h^{-1}, using a flow of 20 vol% C_2H_6/N_2. Before the EDH test, the catalyst was heated to 600 °C in N_2, then switched to the reaction gas flow.

The feed components and the reaction products were analyzed using an on-line gas chromatograph (Agilent 6890) equipped with PLOT/Q (for CO_2), AL/S (for hydrocarbons), and Molsieve 5A (for H_2, O_2, N_2, and CO) columns connected to flame ionization and thermal conductivity detectors, respectively.

Equations (1)-(3) were used for calculating the initial rate of ethene formation (r(C_2H_4)), ethane conversion (X(C_2H_6)) and selectivity to gas-phase products (S_i), respectively. The space-time yield of ethene (STY(C_2H_4), $kg_{C_2H_4}$ kg^{-1}_{cat} h^{-1}) was calculated using equation (4).

$$r(C_2H_4)= \frac{\dot{n}^{out}_{C_2H_4}}{m_{cat}} \quad (1)$$

$$X(C_2H_4)= \frac{\dot{n}^{in}_{C_2H_6}-\dot{n}^{out}_{C_2H_6}}{\dot{n}^{in}_{C_2H_6}} \quad (2)$$

$$S_i=\frac{v_{C_2H_6}}{v_i}\times \frac{\dot{n}^{out}_i}{\dot{n}^{in}_{C_2H_6}-\dot{n}^{out}_{C_2H_6}} \quad (3)$$

$$STY(C_2H_4) = \frac{\dot{n}_{C_2H_4}\times M_{C_2H_4}\times 60}{1000\times m_{cat}} \quad (4)$$

where $\dot{n}$ ($\dot{n}_i$ or $\dot{n}_{C_2H_4}$) with superscripts in or out stands for the molar flows of gas-phase components at the reactor inlet or outlet. v_i is the stoichiometric coefficient for product i. N_2 was used as internal standard to consider reaction-induced changes in the number of moles.

Using the present and previously reported experimental data, i.e., reaction temperature, feed composition, and total pressure (Table S3), we calculated equilibrium ethane conversion ($X(C_2H_6)_{eq}$) using the Cantera package[5] together with the NASA thermodynamic database.[6] The obtained $X(C_2H_6)_{eq}$ values were used to calculate the ratio of $X(C_2H_6)_{exp}/X(C_2H_6)_{eq}$ for a fair comparison of different catalysts in terms of their productivity determined under different reaction conditions.

Computational methods

All spin-polarized density functional theory (DFT) calculations were performed using the Vienna ab initio simulation package (VASP).[7, 8] To accurately treat the Co 3d orbitals, a correction for Coulomb and exchange interactions (DFT+U) was employed by setting U_{eff} = 4.1 eV (U_{eff} = coulomb U – exchange J) applied to the Co 3d states, according to Liu et al. and Sautet et al.[9, 10] The D3 correction method (DFT-D3) was employed in order to include van der Waals (vdW) interactions.[11] The projector-augmented wave (PAW) method was used to represent core-valence interactions.[12] Valence electrons were described by a plane wave basis with an energy cutoff of 480 eV. The generalized gradient approximation with the Perdew-Burke-Ernzerhof (GGA-PBE) functional was used to model electronic exchange and correlation.[13] Electron smearing was employed via Gaussian smearing method with a smearing width consistent to 0.05 eV. The conjugate gradient algorithm was used in geometry optimization calculations. Optimized structures were obtained by minimizing the forces on each ion until they were less than 0.02 eV/Å. The energy convergence criterium was set to 10^{-6} eV. The Brillouin zone was sampled at the 3×3×3 k-point mesh. Transition states (TSs) of each elementary step in the course of the EDH reaction were located using the climbing-image nudged elastic band (CI-NEB) method.[14, 15] The optimized transition states were further verified as having a single imaginary frequency. The structures of cobalt in 6-membered ring and 8-menbered ring of SSZ-13 were modeled according to Verma et al.'s and Schneider et al. [16, 17]

The Gibbs free energy for gaseous species was calculated according to equation (5):

$$G = E_{DFT} + E_{ZPE} + \left[H\left(T, P^{\theta}\right) - H\left(0K, P^{\theta}\right)\right] - T\left[S\left(T, P^{\theta}\right) - S\left(0K, P^{\theta}\right)\right] + k_B T \ln \frac{P}{P^{\theta}} \qquad (5)$$

The Gibbs free energy for surface adsorbed species was calculated according to the following equations:

$$G = E_{DFT} + E_{ZPE} + U^{o} - TS \qquad (6)$$

$$U^{o} = k_B T \sum \frac{hv_i/k_B T}{e^{hv_i/k_B T} - 1} \qquad (7)$$

$$S = k_B \sum \left[\frac{hv_i/k_B T}{e^{hv_i/k_B T} - 1} - \ln\left(1 - e^{-hv_i/k_B T}\right)\right] \qquad (8)$$

where E_{DFT} and E_{ZPE} are DFT-calculated energy and zero-point energy, respectively, enthalpy $H(T, P^{\theta})$ and entropy $S(T, P^{\theta})$ of gaseous molecule were obtained from the JANAF thermochemical tables, P is the partial pressure of gaseous molecule, P^{θ} is the standard pressure (100 kPa), k_B is the Boltzmann constant, h is the Planck constant, T represents the Kelvin temperature, and v_i is the vibrational frequency.

In calculating the Gibbs free energy, the temperature T was set to 873.15 K according to our experimental conditions, and the partial pressures of C_2H_6, C_2H_4, and H_2 were calculated based on the gas-phase composition in our catalytic tests.

Supporting Figures

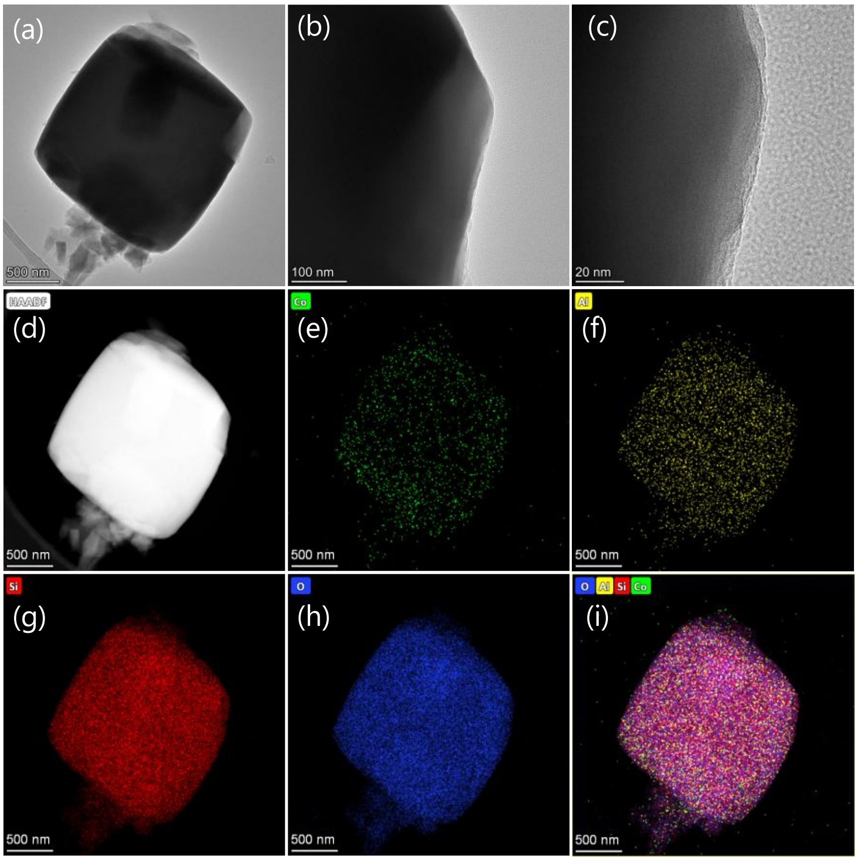

Figure S1. (a-c) HRTEM images and (d-i) EDS mapping of the 0.9Co catalyst.

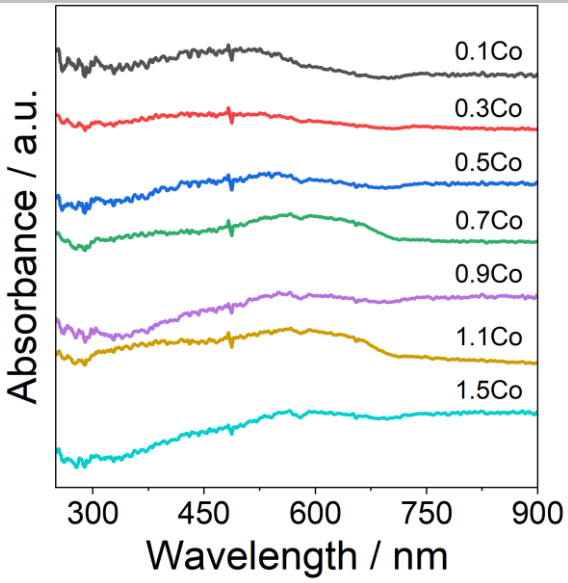

Figure S2. The UV-Vis spectra of differently loaded Co/SSZ-13. The numbers before Co stand for the weight percentage of this metal in the catalysts.

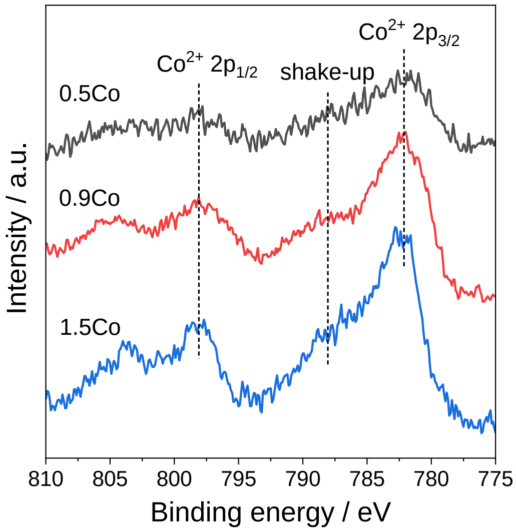

Figure S3. XP spectra of Co 2*p* of differently loaded Co/SSZ-13. The numbers before Co stand for the weight percentage of this metal in the catalysts.

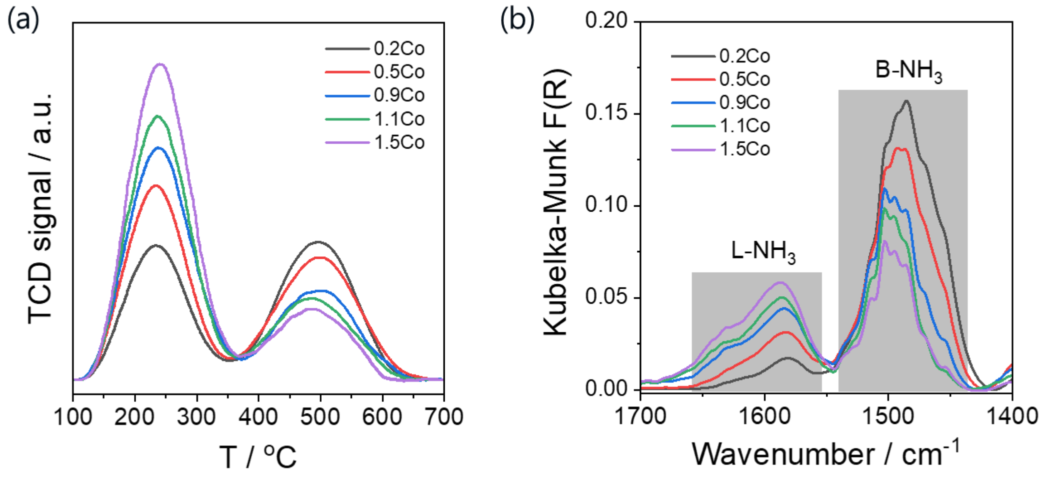

Figure S4. NH_3-TPD profiles and NH_3-DRIFTS spectra (1700-1400 cm^{-1}) of differently loaded Co/SSZ-13. The numbers before Co stand for the weight percentage of this metal in the catalysts.

As shown in Figure S4(a), the NH_3-TPD profiles of Co/SSZ-13 catalysts exhibit two well-resolved desorption maxima, corresponding to acid sites of different strengths. The low-temperature peak is primarily attributed to NH_3 desorption from Lewis acid sites, whereas the high-temperature peak arises mainly from Brønsted acid sites.[12] With increasing Co loading, the intensity of the Lewis-related peak increases, while that of the Brønsted-related peak decreases. This evolution is fully consistent with the NH_3-DRIFTS spectra in the 1700–1400 cm^{-1} region (Figure S4(b)), where the bands of NH_3 coordinated to Lewis sites intensify at higher Co contents, accompanied by a progressive attenuation of Brønsted-related features.

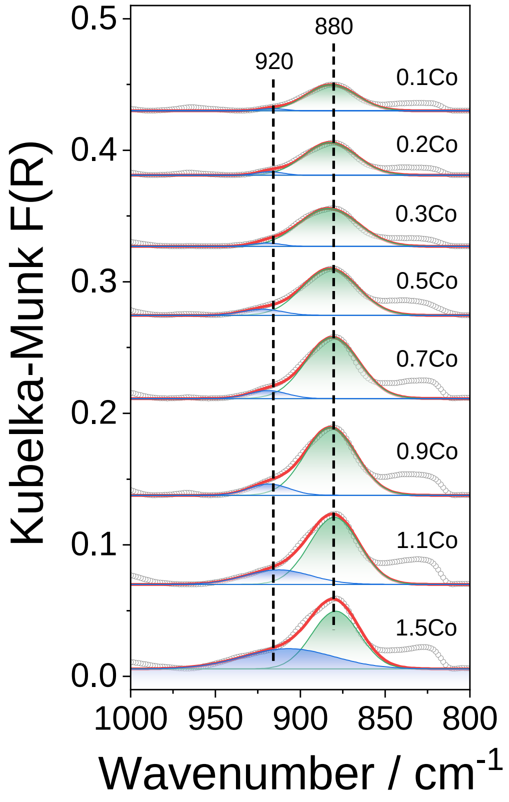

Figure S5. Gaussian fitting of the NH_3-DRIFTS spectra of differently loaded Co/SSZ-13. The numbers before Co stand for the weight percentage of this metal in the catalysts.

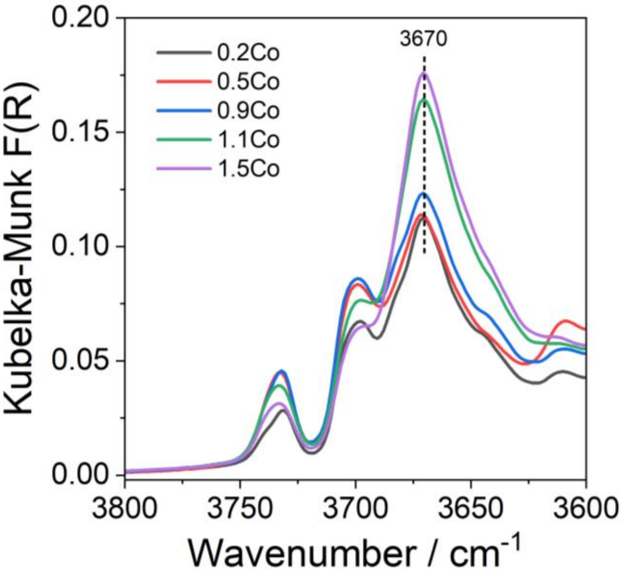

Figure S6. NH_3-DRIFTS spectra of differently loaded Co/SSZ-13 recorded in the *v*(OH) region (3800–3600 cm^{-1}) for the different Co loading catalysts. The numbers before Co stand for the weight percentage of this metal in the catalysts.

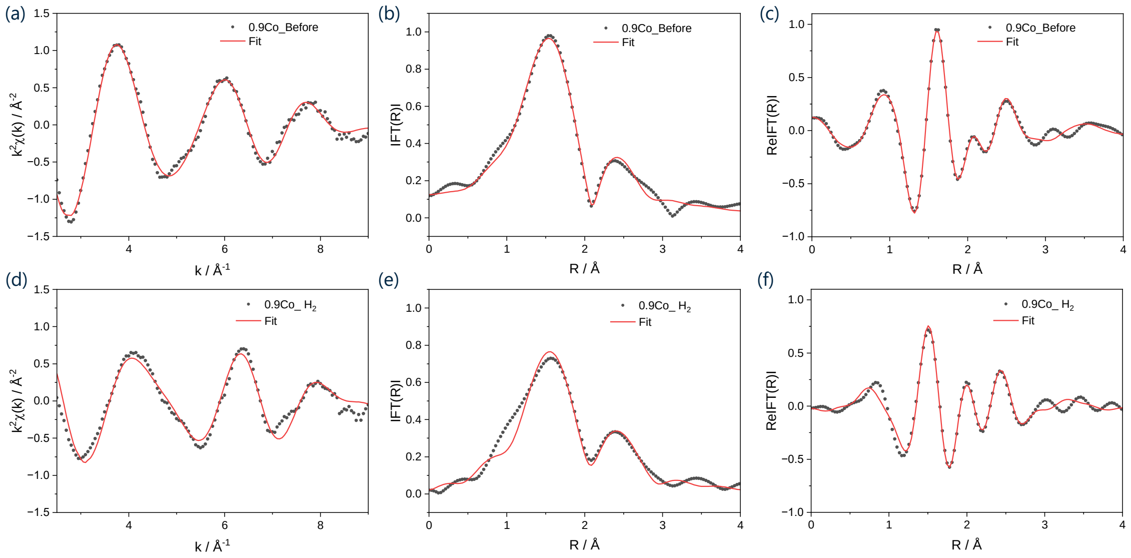

Figure S7. Co K-edge k^2-weighted (a, d) χ(k) EXAFS function and its Fourier transform (b, e) magnitude and (c, f) real part in the R-space of the 0.9Co sample, and the corresponding fits for (a-c) 0.9Co_Before and (d-f) 0.9Co _H_2.

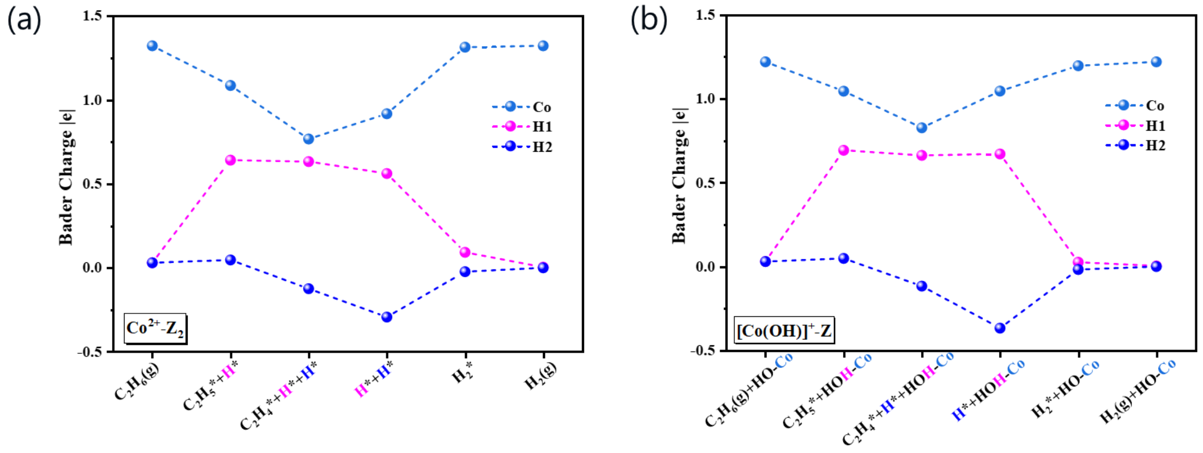

Figure S8. Bader charge evolution along the EDH pathway over (a) Co^{2+}–Z_2 and (b) $[Co(OH)]^{+}$–Z sites.

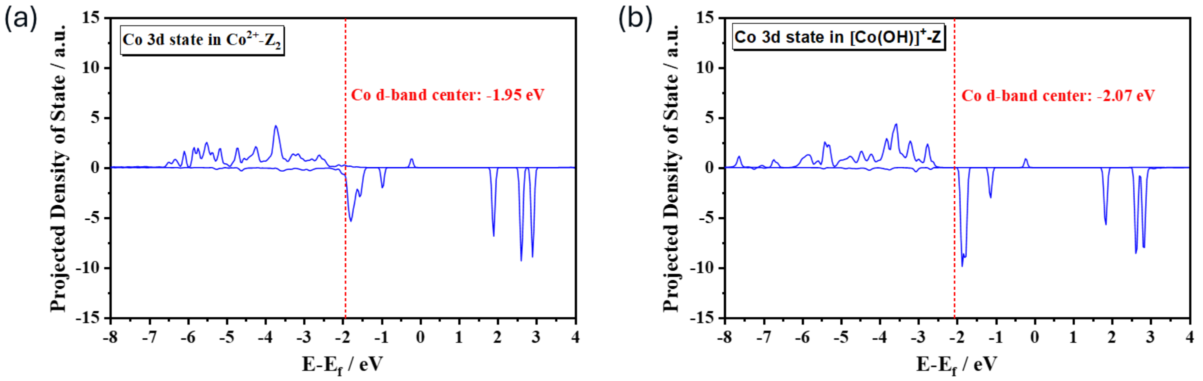

Figure S9. Projected density of states (PDOS) of Co 3d orbitals for (a) Co^{2+}–Z_2 and (b) $[Co(OH)]^+$–Z sites in SSZ-13.

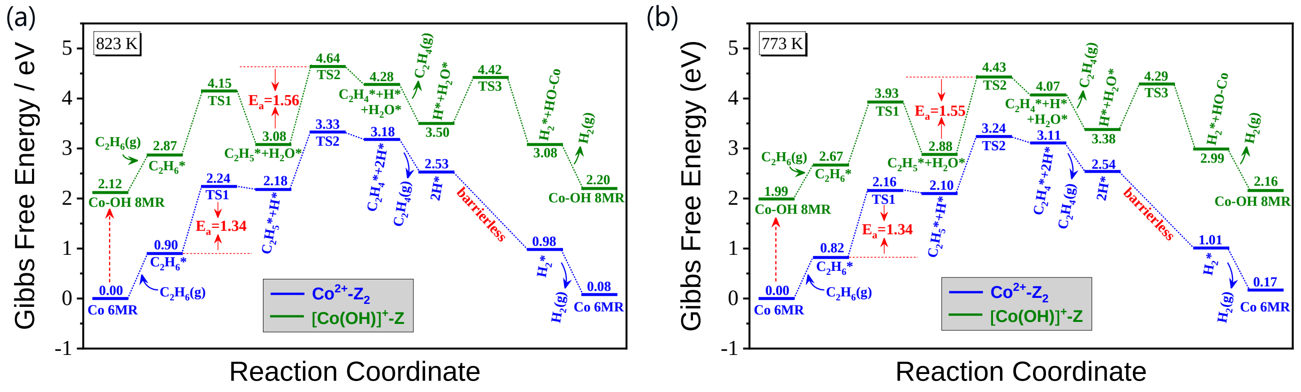

Figure S10. Gibbs free-energy profiles for ethane dehydrogenation over Co^{2+}–Z_2 and $[Co(OH)]^+$–Z sites under different conditions: (a) 823 K, 1 atm; (b) 773 K, 1 atm.

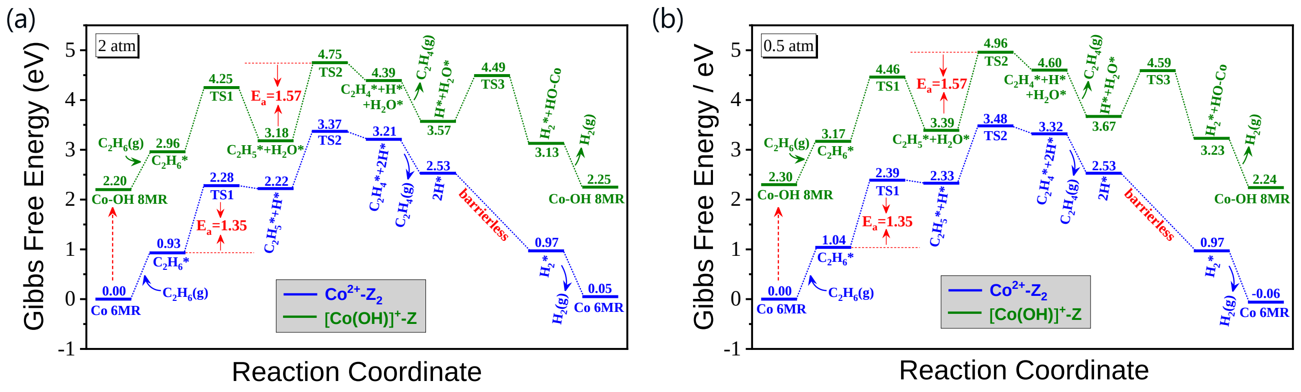

Figure S11. Gibbs free-energy profiles for ethane dehydrogenation over Co^{2+}–Z_2 and $[Co(OH)]^+$–Z sites under different conditions: (a) 873 K, 2 atm; (b) 873 K, 0.5 atm.

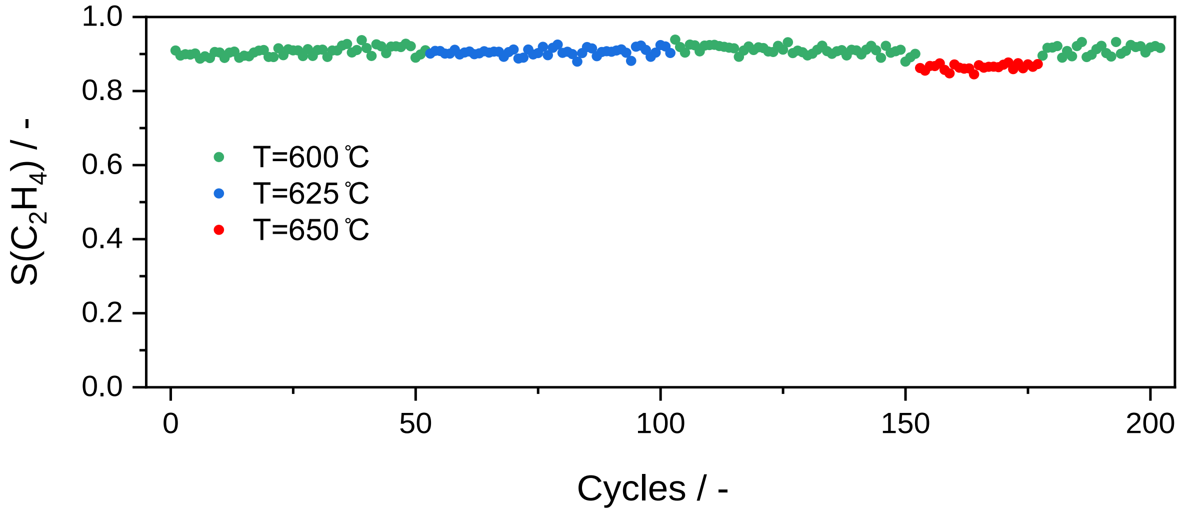

Figure S12. On-stream profiles of $S(C_2H_4)$ over 0.9Co in a series of 200 EDH/regeneration cycles at 600°C (green), 625°C (blue) and 650°C (red).

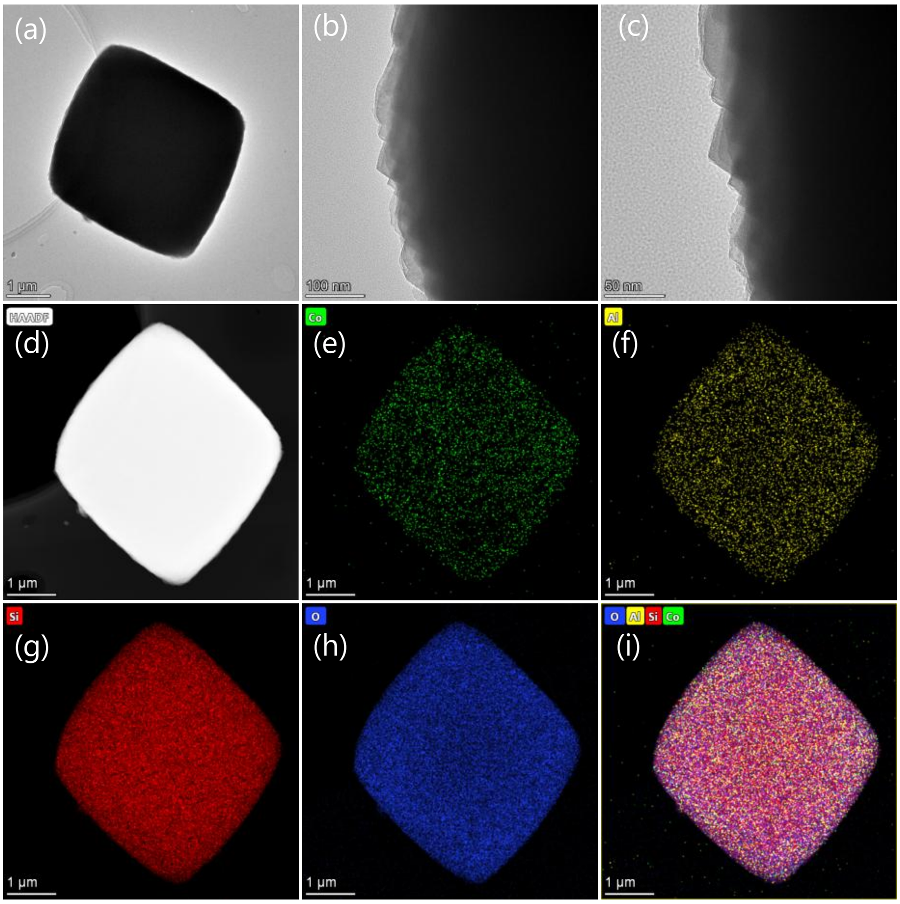

Figure S13. (a-c) HRTEM image and (d-i) EDS mapping of spent 0.9Co after 200 EDH/regeneration cycles.

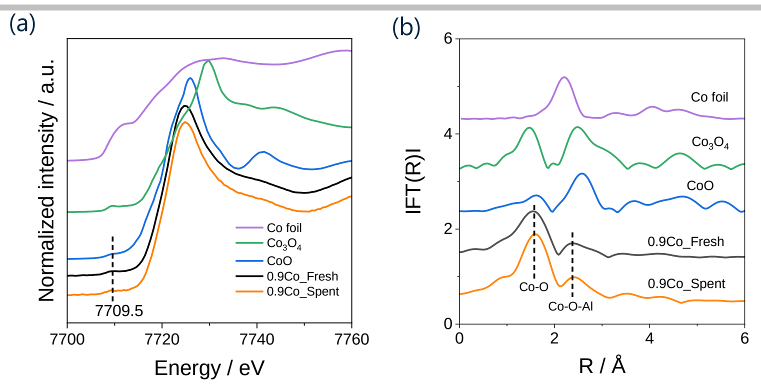

Figure S14. (a) The Co K-edge XANES of 0.9Co_Fresh, 0.9Co_Spent and reference samples (CoO, Co_3O_4 and Co foil); (b) The corresponding k^2-weighted Fourier transform spectra.

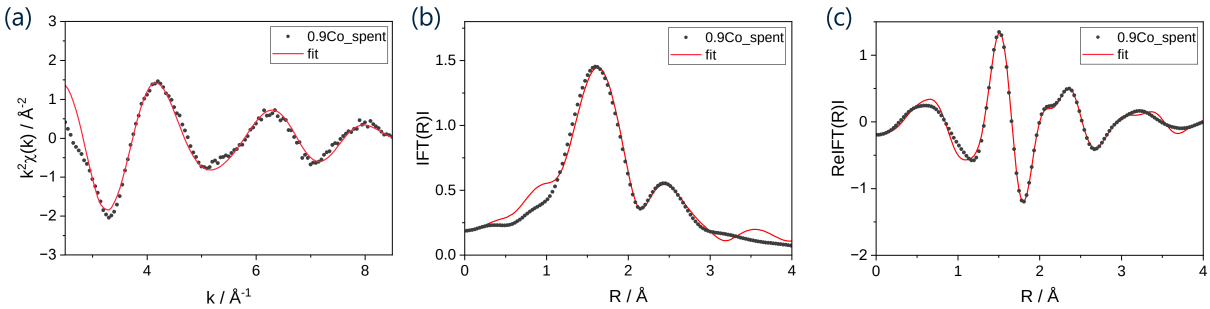

Figure S15. Co K-edge k^2-weighted (a) χ(k) EXAFS function and its Fourier transform (b) magnitude and (c) real part in the R-space of the 0.9Co_Spent.

Supporting Tables

Table S1. Brunauer-Emmett-Teller (BET) specific surface area and relative distribution of Co^{2+} species obtained from XPS curve fitting shown in Figure S5.

Sample	S_{BET}/m^2 g^{-1}	Relative distribution of Co^{2+} species	
		Cu^{2+}-Z_2 (880 cm^{-1})/%	$[Cu(OH)]^+$-Z (920 cm^{-1})/%
0.1Co	579.2	98.03	1.97
0.2Co	586.3	97.00	3.00
0.3Co	573.4	95.83	4.17
0.5Co	571.6	92.21	7.79
0.7Co	576.4	91.58	8.48
0.9Co	568.9	88.47	11.53
1.1Co	575.5	76.86	23.14
1.5Co	563.8	59.77	40.23

Table S2. Fit parameters of Co K-edge EXAFS. The fitting of Fourier-transformed $k^{1,2}$-weighted data was performed in the k-range of [3.0-9.0] and r-range of [1.0–3.0] Å. The amplitude reduction factor S_0^2=0.76 was obtained from the fitting the Co foil reference and fixed for further fits.

Sample	Type of scattering	R/ Å	σ^2, Å^2	CN	ΔE_0, eV	R_f, %
0.9Co_Before	Co-O	2.00 (1)	0.004 (1)	3.5 (1)	-1.4 (7)	0.3
	Co-O	2.16 (1)	0.004 (1)	3.2 (1)		
	Co-O-Al	2.81 (1)	0.009 (1)	2.1 (2)		
0.9Co_H_2	Co-O	2.02 (1)	0.006 (1)	3.6 (2)	3.6 (5)	1.2
	Co-O-Al	2.80 (1)	0.005 (2)	2.1 (1)		
0.9Co_Spent	Co-O	1.99(5)	0.003(1)	3.3(2)	3.2(8)	0.9
	Co-O	2.12(3)	0.003(1)	2.7(3)		
	Co-O-Al	2.92(2)	0.005(2)	2.3(4)		

Table S3. Catalytic performance (ethane conversion ($X(C_2H_6)_{exp}$), the ratio of the experimentally determined conversion to the corresponding equilibrium one ($X(C_2H_6)_{exp}/X(C_2H_6)_{eq}$), space time yield of ethene formation ($STY(C_2H_4)$)) of various reported catalysts from literature and from the present study in the non-oxidative ethane dehydrogenation to ethylene under different reaction conditions (temperature (T), and weight hourly space velocity (WHSV)).

Catalyst	T/°C	$X(C_2H_6)_{exp}$/%	$X(C_2H_6)_{eq}$/%	$X(C_2H_6)_{exp}/X(C_2H_6)_{eq}$	WHSV/h^{-1}	$STY(C_2H_4)$/kg $h^{-1}\cdot kg^{-1}$	Ref.
5mol%Ga_2O_3/Al_2O_3	650	28	52	0.53	2.0	0.48	[18]
Cr_2O_3/Oxidized diamond	650	7	52	0.13	2.0	0.13	[19]
Cr/MgAlOx	700	22	60	0.36	5.4	0.88	[20]
5.0Cr–10Ce/SBA-15	700	41	63	0.65	1.2	0.38	[21]
Cr_2O_3/Al_2O_3	600	13	23	0.59	12.6	1.41	[22]
Pt–In(0.7)/SiO_2	600	15	30	0.50	3.0	0.42	[23]
Ga/TiSi-15	650	46	79	0.58	0.4	0.13	[24]
2.5P-Mo/ZSM-5	600	16	27	0.60	0.7	0.07	[25]
10Fe/ZSM-5	600	22	28	0.79	4.3	0.63	[26]

Catalyst	T/°C	$X(C_2H_6)_{exp}$/%	$X(C_2H_6)_{eq}$/%	$X(C_2H_6)_{exp}/X(C_2H_6)_{eq}$	WHSV/h^{-1}	$STY(C_2H_4)$/kg $h^{-1}\cdot kg^{-1}$	Ref.
Ni_3Ga/Al_2O_3(1:1)	600	15	27	0.55	1.6	0.21	[27]
Pt-ISAS@NaY	700	27	66	0.41	4.0	1.00	[28]
c-Pt_3In/SiO_2	600	36	46	0.79	2.0	0.62	[29]
Au/TiSi-20	650	16	79	0.20	0.4	0.05	[30]
In-CHA	650	26	61	0.43	0.8	0.19	[31]
$PtZn_2/Al_2O_3$	600	19	32	0.60	1.2	0.21	[32]
FeS-1-EDTA	600	26	28	0.94	0.8	0.19	[33]
0.8Cr/MFI	650	28	49	0.56	1.6	0.41	[34]
Pt/M-TS-1 (EA)	600	7	19	0.34	12.1	0.72	[35]
Co/SAPO-34-IE	600	8	18	0.43	3.6	0.26	[36]
1Fe/HZ5-HTS	650	53	72	0.75	0.4	0.15	[37]

Catalyst	T/°C	$X(C_2H_6)_{exp}$/%	$X(C_2H_6)_{eq}$/%	$X(C_2H_6)_{exp}/X(C_2H_6)_{eq}$	WHSV/h^{-1}	$STY(C_2H_4)$/kg $h^{-1}\cdot kg^{-1}$	Ref.
EA-0.2Co/SiO_2-pretreated	625	35	67	0.52	1.8	0.59	[38]
0.5Co@MFI	650	26	49	0.53	1.6	0.39	[39]
6%Co@MFI	600	16	18	0.91	7.2	1.08	[40]
Co/S-1	590	13	17	0.77	7.1	0.75	[41]
PSC α-Al_2O_3	700	34	76	0.44	1.6	0.49	[42]
$PtIn_2/SiO_2$	600	36	54	0.67	2.7	0.90	[43]
Ca-PtIn2/SiO_2	600	12	54	0.22	2.7	0.30	[43]
1Pt0.7Zn@S-1	550	16	16	1.00	1.0	0.14	[44]
Ir1/N-C	600	15	17	0.88	10.7	1.42	[45]
PtIn-MFI	600	32	35	0.91	3.7	1.09	[46]
PtIn-MFI	650	45	52	0.86	3.7	1.53	[46]

Catalyst	T/°C	$X(C_2H_6)_{exp}$/%	$X(C_2H_6)_{eq}$/%	$X(C_2H_6)_{exp}/X(C_2H_6)_{eq}$	WHSV/h^{-1}	$STY(C_2H_4)$/kg $h^{-1}\cdot kg^{-1}$	Ref.
0.04Pt-3Ga-ZSM5	600	24	42	0.57	2.0	0.50	[47]
PtZn@S-1−170 (0.4 wt % Pt)	550	9	9	0.96	6.0	0.34	[48]
Co@Deal BEA	600	24	30	0.81	9.1	2.08	[49]
Co0.2%@0.01W-S-1	600	23	30	0.76	4.5	1.04	[50]
0.9Co	600	14	32	0.46	24.1	3.1	This work
0.9Co	600	19	32	0.60	12.1	2.0	This work
0.9Co	600	28	32	0.90	5.1	1.2	This work

Supporting References

[1] T. Zhang, Y. Qiu, G. Liu, J. Chen, Y. Peng, B. Liu, J. Li, *J. Catal.* **2020**, *392*, 322-335.

[2] V. Fridman (Sud-Chemie Inc.), US 8101541 B2, **2012**.

[3] A. Iglesias-Juez, A. M. Beale, K. Maaijen, T. C. Weng, P. Glatzel, B. M. Weckhuysen, *J. Catal.* **2010**, *276*, 268-279.

[4] B. Ravel, M. Newville, *J. Synchrotron Radiat.* **2005**, *12*, 537-541.

[5] Cantera, Version 2.6.0, https://cantera.org.

[6] B. McBride, S. Gordon, M. Reno, *NASA Technical Memorandum 4513: Coefficients for Calculating Thermodynamic and Transport Properties of Individual Species*; NASA, 1993.

[7] G. Kresse, J. Furthmüller, *Phys. Rev. B.* **1996**, *54*, 11169.

[8] G. Kresse, J. Furthmüller, *Comput. Mater. Sci.* **1996**, *6*, 15-50.

[9] B. Liu, Y. Zha, L. Xin, H. Zhang, Y. Xu, X. Liu, *J. Catal.* **2024**, *430*, 115307.

[10] K.-W. Park, A. M. Kolpak, *J. Catal.* **2018**, *365*, 115-124.

[11] S. Grimme, J. Antony, S. Ehrlich, H. Krieg, *J. Chem. Phys.* **2010**, *132*, 154104

[12] P. E. Blöchl, *Phys. Rev. B* **1994**, *50*, 17953.

[13] J. P. Perdew, K. Burke, M. Ernzerhof, *Phys. Rev. Lett.* **1996**, *77*, 3865.

[14] Henkelman, H. Jónsson, *J. Chem. Phys.* **2000**, *113*, 9978-9985.

[15] G. Henkelman, B. P. Uberuaga, H. Jónsson, *J. Chem. Phys.* **2000**, *113*, 9901-9904.

[16] A. A. Verma, S. A. Bates, T. Anggara, C. Paolucci, A. A. Parekh, K. Kamasamudram, A. Yezerets, J. T. Miller, W. N. Delgass, W. F. Schneider, *J. Catal.* **2014**, *312*, 179-190.

[17] C. Paolucci, A. A. Verma, S. A. Bates, V. F. Kispersky, J. T. Miller, R. Gounder, W. N. Delgass, F. H. Ribeiro, W. F. Schneider, *Angew. Chem. Int. Ed.* **2014**, *53*, 11828-11833.

[18] K. Nakagawa, C. Kajita, Y. Ide, M. Okamura, S. Kato, H. Kasuya, N. O. Ikenaga, T. Kobayashi, T. Suzuki, *Catal. Lett.* **2000**, *64*, 215-221.

[19] K. Nakagawa, C. Kajita, N.-O. Ikenaga, T. Suzuki, T. Kobayashi, M. Nishitani-Gamo, T. Ando, *J. Phys. Chem. B.* ***2003***, *107*, 4048-4056.

[20] A. Tsyganok, P. J. Harlick, A. Sayari, *Catal. Commun.* **2007**, *8*, 850-854.

[21] X. Shi, S. Ji, K. Wang, *Catal. Lett.* **2008**, *125*, 331-339.

[22] D. Shee, A. Sayari, *Appl. Catal. A.* **2010**, *389*, 155-164.

[23] E. C. Wegener, Z. Wu, H.-T. Tseng, J. R. Gallagher, Y. Ren, R. E. Diaz, F. H. Ribeiro, J. T. Miller, *Catal. Today.* **2018**, *299*, 146-153.

[24] T.-Q. Lei, Y.-H. Cheng, C.-X. Miao, W.-M. Hua, Y.-H. Yue, Z. Gao, *Fuel Process. Technol.* **2018**, *177*, 246-254.

[25] Z. Ji, H. Lv, X. Pan, X. Bao, *J. Catal.* **2018**, *361*, 94-104.

[26] L.-C. Wang, Y. Zhang, J. Xu, W. Diao, S. Karakalos, B. Liu, X. Song, W. Wu, T. He, D. Ding, *Appl. Catal. B Environ.* **2019**, *256*, 117816.

[27] Y. He, Y. Song, S. Laursen, *ACS Catal.* **2019**, *9*, 10464-10468.

[28] Y. Liu, Z. Li, Q. Yu, Y. Chen, Z. Chai, G. Zhao, S. Liu, W.-C. Cheong, Y. Pan, Q. Zhang, *J. Am. Chem. Soc.* **2019**, *141*, 9305-9311.

[29] N.J. Escorcia, N.J. LiBretto, J.T. Miller, C.W. Li, *ACS Catal.* **2020**, *10*, 9813-9823.

[30] Q. Xie, T. Lei, C. Miao, W. Hua, Y. Yue, Z. Gao, *Catal. Lett.* **2020**, *150*, 2013-2020.

[31] Z. Maeno, S. Yasumura, X. Wu, M. Huang, C. Liu, T. Toyao, K.-i. Shimizu, *J. Am. Chem. Soc.* **2020**, *142*, 4820-4832.

[32] X. Li, Y. Zhou, B. Qiao, X. Pan, C. Wang, L. Cao, L. Li, J. Lin, X. Wang, *J. Energy Chem.* **2020**, *51*, 14-20.

[33] Z. Yang, H. Li, H. Zhou, L. Wang, L. Wang, Q. Zhu, J. Xiao, X. Meng, J. Chen, F.-S. Xiao, *J. Am. Chem. Soc.* **2020**, *142*, 16429-16436.

[34] S. De, S. Ould-Chikh, A. Aguilar, J.-L. Hazemann, A. Zitolo, A. Ramirez, S. Telalovic, J. Gascon, *ACS Catal.* **2021**, *11*, 3988-3995.

[35] Y. Pan, A. Bhowmick, W. Wu, Y. Zhang, Y. Diao, A. Zheng, C. Zhang, R. Xie, Z. Liu, J. Meng, *ACS Catal.* **2021**, *11*, 9970-9985.

[36] Y. Xu, W. Yu, H. Zhang, J. Xin, X. He, B. Liu, F. Jiang, X. Liu, *ACS Catal.* **2021**, *11*, 13001-13019.

[37] L. Wu, Z. Fu, Z. Ren, J. Wei, X. Gao, L. Tan, Y. Tang, *ChemCatChem.* **2021**, *13*, 4019-4028.

[38] K. Yu, S. Srinivas, C. Wang, W. Chen, L. Ma, S. N. Ehrlich, N. Marinkovic, P. Kumar, E. A. Stach, S. Caratzoulas, *ACS Catal.* **2022**, *12*, 11749-11760.

[39] S. De, A. Aguilar-Tapia, S. Ould-Chikh, A. Zitolo, J.-L. Hazemann, G. Shterk, A. Ramirez, J. Gascon, *J. Mater. Chem. A* **2022**, *10*, 9445-9456.

[40] Y. Xu, W. Hu, Y. Li, H. Su, W. Liang, B. Liu, J. Gong, Z. Liu, X. Liu, *ACS Catal.* **2023**, *13*, 1830-1847.

[41] L. Liu, H. Li, H. Zhou, S. Chu, L. Liu, Z. Feng, X. Qin, J. Qi, J. Hou, Q. Wu, *Chem.* **2023**, *9*, 637-649.

[42] X. Li, W. Li, J. Zhang, W. Yin, Y. Xia, K. Xie, *Angew. Chem. Int. Ed.* **2024**, *136*, e202315274.

[43] V. Yadav, J. M. Rosenberger, B. K. Bolton, R. Gounder, C. W. Li, *J. Catal.* **2024**, *432*, 115446.

[44] Y. Zhang, W. Wang, L. Zhou, Y. Zhang, *Appl. Surf. Sci.* **2024**, *648*, 159099.

[45] Y. Li, C.-Q. Xu, C. Chen, Y. Zhang, S. Liu, Z. Zhuang, Y. Zhang, Q. Zhang, Z. Li, Z. Chen, *J. Am. Chem. Soc.* **2024**. 146. 20668-20677.

[46] C. Zhu, W. Li, T. Chen, Z. He, E. Villalobos, C. Marini, J. Zhou, B. T. Woon Lo, H. Xiao, L. Liu, *Angew. Chem. Int. Ed.* **2024**, *136*, e202409784.

[47] X. Dou, K. Li, K. Zhang, C. Zhu, D.M. Meira, Y. Song, P. He, L. Zhang, L. Liu, *JACS Au.* **2024**, *4*, 3547-3557.

[48] L. Zhang, J. Tian, R. Liu, B. Ma, C. Zhao, *ACS Catal.* **2025**, *15*, 10971-10981.

[49] A. Bhowmick, S. Srinivas, J. Zhang, J. Moncada, C.J. Titus, B. Ravel, C. Jaye, D. A. Fischer, G. Yarema, S. Luo, *ACS Catal.* **2025**, *15*, 10372-10390.

[50] X. Yin, T. Zhang, Y. Ma, Q. Sun, *J. Energy Chem.* **2025**, *103*, 525-534.
